# Risk factors for healthcare-associated infection among children in a low-and middle-income country

**DOI:** 10.1186/s12879-022-07387-2

**Published:** 2022-04-26

**Authors:** Indah K. Murni, Trevor Duke, Sharon Kinney, Andrew J. Daley, Muhammad Taufik Wirawan, Yati Soenarto

**Affiliations:** 1 Department of Child Health, Dr. Sardjito Hospital, Faculty of Medicine, Public Health and Nursing, Universitas Gadjah Mada, Jalan Kesehatan No 1, Sekip, Yogyakarta, 55281 Indonesia; 2 Centre for Child Health-Pediatric Research Office (CCH-PRO), Faculty of Medicine, Public Health and Nursing, Universitas Gadjah Mada, Yogyakarta, Indonesia; 3 Centre for International Child Health, University of Melbourne, MCRI, Melbourne, VIC Australia; 4 Pediatric Intensive Care Unit, Royal Children’s Hospital, Melbourne, VIC Australia; 5 Department of Pediatrics, University of Melbourne, Melbourne, VIC Australia; 6 Department of Pediatrics and Nursing, University of Melbourne, Royal Children’s Hospital, Melbourne, VIC Australia; 7 Laboratory Services, Infection Prevention and Control, Royal Children’s Hospital, Melbourne, VIC Australia

**Keywords:** Risk factor, Healthcare-associated infection, Nosocomial infection, Children, Low- and middle-income countries, Indonesia

## Abstract

**Background:**

Healthcare-associated infections (HAI) are one of significant causes of morbidity and mortality. Evaluating risk factors associated with HAI are important to improve clinical outcomes. We aimed to evaluate the risk factors of HAI in children in a low-to middle-income country.

**Methods:**

A prospective cohort study was conducted during 43 months at a teaching hospital in Yogyakarta, Indonesia. All consecutive patients admitted to pediatric ICU and pediatric wards > 48 h were eligible. Those eligible patients were observed daily to identify the presence of HAI based on CDC criteria. The risk factors of HAI were identified. Multivariable logistic regression was used to identify independent risk factors.

**Results:**

Total of 2612 patients were recruited. Of 467 were diagnosed as HAI. The cumulative incidence of HAI was 17.9%. In the multivariable analysis; length of stay > 7 days, severe sepsis, use of urine catheter, central venous catheter (CVC), non-standardized antibiotics, and aged < 1 year were independently associated with increased risk of HAI with adjusted OR (95%CI): 5.6 (4.3–7.3), 1.9 (1.3–2.9), 1.9 (1.3–2.6), 1.8 (1.1–2.9), 1.6 (1.2–2.0), and 1.4 (1.1–1.8), respectively.

**Conclusions:**

This study found that length of stay > 7 days, use of urine catheter and CVC, non-standardized antibiotic use, aged < 1 year, and had a diagnosis of severe sepsis increased risk of HAI.

**Supplementary Information:**

The online version contains supplementary material available at 10.1186/s12879-022-07387-2.

## Background

Hospitalized children are at risk of developing healthcare-associated infections (HAI) because of intrinsic factors such as young age, severity of the disease, and low nutritional status; and also their extrinsic factors such as the presence of multi-resistant bacteria in the health care environment, the use of invasive devices such as central line catheter, mechanical ventilation and urinary catheter [1, 2]. Knowing risk factors for developing healthcare-associated infections in hospitalized children in settings with limited resources is important when there is a high burden of healthcare-associated infections [2]. Furthermore, determining the risk factors of HAI is the most importance to identify strategies to prevent the occurrence of healthcare-associated infections.

A published review has systematically collated risk factors of healthcare-associated infection, but focused on patients admitted to the pediatric intensive care unit (PICU) and involved both high- and low- and middle-income countries [1]. Some studies have also identified factors to predict the development of HAI in low- and middle-income countries; however, most of them were undertaken in neonates and intensive care unit settings [3–10]. Moreover, most published studies on risk factors of HAI focused on specific types of healthcare-associated infection such as central line-associated bloodstream infection and ventilator-associated pneumonia [6–8, 10]. Few studies have explored the risk factors of overall healthcare-associated infection in the pediatric wards and PICU in low- and middle-income countries [1]. Therefore, we aimed to explore the independent risk factors of HAI in children in our setting.

## Methods

### Study population, time and setting

A prospective cohort study was conducted in the PICU and general pediatric wards at Dr Sardjito Hospital in Yogyakarta, Indonesia during 1st June 2016 to 31st December 2019 [11]. The Dr. Sardjito Hospital is a referral hospital for Yogyakarta and the Southern part of the Central Java provinces in Indonesia, and provides services to a population of approximately 3.5 million people. The public general pediatric wards consist of infectious and non-infectious wards. There are 39 beds and there are multiple beds in a room, and approximately 1500 children are admitted to these wards annually. The PICU has nine beds, and around 320 patients are admitted each year. During the follow up of all recruited patients, factors associated with the risk of developing HAI were identified.

### Data collection

The definitions of HAI were based on the US Centers for Disease Control and Prevention (CDC) National Healthcare Safety Network (NHSN) and the National Nosocomial Infections Surveillance (NNIS) system. Every child in the study was observed each day to see whether he/she fulfilled the CDC criteria for a HAI [11, 12]. Investigations of the causes of fever and other signs of infection were at the discretion of the treating clinical staff. If clinical criteria for suspected HAIs were fulfilled and the child had not been investigated by the treating doctors, the clinical staff was advised, so they could collect a culture of blood, urine or other sterile sites, as appropriate, on the same day. Once a nosocomial infection was identified according to the definition shown above, the specific type of infection was determined [11, 12].

The infection control consists of infectious disease doctors, specialist doctors, nurses, the pharmacist and clinical pathology and microbiology staff. We have no protected time for infection control and antibiotic stewardship nor do health care professionals involved receive additional salary for these roles [11]. There are established IPC practices and protocols including measures to prevent nosocomial bloodstream infections, ventilator-associated pneumonia and catheter-associated urinary tract infections. The audit was done and fed back to the health workers, however these were not routinely done.

The risk factors of healthcare-associated infection were identified based on epidemiological and clinical parameters. The following were evaluated as potential risk factors: age less than 12 months, malnutrition, immunocompromised condition, presence of genetic syndrome, referred from another hospital, use of central venous catheter, use of mechanical ventilation, use of urinary catheter, length of hospital stay more than 7 days, exposure to non-standardized antibiotic therapy according to WHO guidelines, recent surgery, and severe sepsis or septic shock.

Malnutrition with severe wasting was defined by weight-for-height < −3 SD using Z score. Immunocompromised condition was defined when the underlying diseases of patients were malignancy or HIV/AIDS. Presence of genetic syndrome was defined when patients were diagnosed as having any congenital dysmorphic syndrome. Recent surgery was defined when patients underwent surgery within 30 days at the time of admission or during the hospital stay.

Whether antibiotic use was standardised or non-standardised was assessed at the time of study entry in every patient with a community-acquired infection and was treated with antibiotics [1, 11]. The standards for empirical antibiotic prescribing for community-acquired infections were based on the recommendations contained in the WHO Pocket Book of Hospital Care for Children [13].

### Outcome measures

The risk factors of HAI were defined as the significant risk factors of healthcare-associated infection derived from multivariable logistic regression analysis.

### Data analysis

A univariable analysis was done to determine the significance and strength of the association between each factor and the development of healthcare-associated infection. We assessed significance by the x^2^ for all categorical variables and p < 0.05 was considered to indicate statistical significance. Multivariable logistic regression analysis was also conducted to determine factors that were associated with healthcare-associated infection. For these analyses, we selected all potential risk factors, including all variables found to be significant in the univariable analysis, and entered them into a multivariable logistic regression analysis. The results of multivariable analysis were reported as odds ratios with their 95% confidence interval.

The multicollinearity analysis including the correlation coefficient, tolerance, and variance inflation factor was also done to determine strong relationship between the independent variables (risk factors). Strong or perfect relationship among independent variables was considered when the tolerance are < 0.10 and variance inflation factor > 10 [14].

## Results

During the study, we recruited 2612 patients. Patients admitted to PICU and pediatric wards were not similar with regard to sex, young age, the underlying diagnosis. Four hundred and sixty-seven developed healthcare-associated infections during the follow-up. Therefore, the cumulative incidence of healthcare-associated infection was 17.9% (467/2612) (Table 1). About 51/2612 (2%) patients developed nosocomial bloodstream infections, nosocomial pneumonia in 94 (3.6%), and ventilator associated pneumonia in 30 (1.1%). The most common organisms were *K. pneumoniae* (22/96, 22.9%)*, E. coli* (12/96, 12.5%)*, Acinetobacter spp* (10/96, 10.4%)*,* and *P. aeruginosa* (6/96, 6.2%).

**Table 1:** Characteristics of children with and without healthcare-associated infections

Baseline characteristics	HAI n = 467 (%)	No HAI n = 2145 (%)	p value
Age, n (%)			
< 12 months old	166 (35.5)	504 (23.5)	< 0.001
12–60 months old	111 (23.8)	528 (24.6)	
> 60–120 months old	63 (13.5)	428 (20.0)	
> 120 months old	127 (27.2)	685 (31.9)	
Sex, n (%)			
Male	226 (48.4)	1058 (49.3)	0.721
Female	241 (51.6)	1087 (50.7)	
Underlying diagnosis, n (%)			
Neurology	115 (24.6)	417 (19.4)	0.013
Nephrology	63 (13.5)	229 (10.7)	0.089
Respiratory	30 (6.4)	150 (7.0)	0.762
Cardiovascular	90 (19.3)	560 (26.1)	0.002
Hematology and oncology	3 (0.6)	28 (1.3)	0.344
Gastrohepatology	61 (13.1)	266 (12.4)	0.700
Infectious	32 (6.9)	218 (10.2)	0.030
Immunology	29 (6.2)	193 (9.0)	0.054
Endocrinology	6 (1.3)	37 (1.7)	0.687
Malnutrition	2 (0.2)	7 (0.3)	0.667
Surgery	36 (7.7)	40 (1.9)	< 0.001

*HAI* healthcare associated infection

We evaluated 13 risk factors associated with healthcare-associated infection among these children. The univariable analysis identified 10 risk factors that were significantly associated with the development of healthcare-associated infection including aged less than 12 months, malnutrition, referred from another hospital, use of central venous catheter, use of mechanical ventilation, use of urinary catheter, length of hospital stays > 7 days, use of non-standardised antibiotics, recent surgery and had severe sepsis or septic shock. In the multivariable analysis, length of hospital stays more than 7 days, use of central venous catheter, use of urinary catheter, use of non-standardized antibiotics, aged < 12 months, and developed severe sepsis or septic shock were independently associated with the development of healthcare-associated infection. Length of hospital stay > 7 days was associated with > 5 times higher in the probability of healthcare-associated infection. Use of central venous catheter, urinary catheter, non-standardized antibiotics, aged < 12 months, and had severe sepsis or septic shock were independently associated with 1.5 to 2 times higher risk of developing healthcare-associated infection (Table 2). In the multicollinearity analysis, we found that the tolerance were > 0.10 and variance inflation factor < 10 for all independent variables.

**Table 2:** Risk factors of healthcare-associated infections

Risk factors	HAI n = 467 (%)	No HAI n = 2145 (%)	Unadjusted OR	p value	Adjusted OR	p value
Length of hospital stay						
> 7 days	386 (82.7)	897 (41.8)	6.6 (5.1–8.6)	< 0.001	5.6 (4.3–7.3)	< 0.001
≤ 7 days	81 (17.3)	1248 (58.2)	1			
Non-standardized antibiotics						
Yes	237 (50.7)	520 (24.2)	3.2 (2.6–4.0)	< 0.001	1.6 (1.2–2.0)	< 0.001
No	230 (49.3)	1625 (75.8)	1			
Presence of genetic syndrome						
Yes	26 (5.6)	87 (4.1)	1.4 (0.9–2.2)	0.166		
No	441 (94.4)	2058 (95.9)	1			
Malnutrition						
Yes	50 (10.7)	134 (6.2)	1.8 (1.3–2.5)	0.001	1.4 (0.9–2.1)	0.067
No	417 (89.3)	2011 (93.8)	1			
Immunocompromised condition						
Yes	114 (24.4)	526 (24.5)	1.0 (0.8–1.3)	1.000		
No	353 (75.6)	1619 (75.5)	1			
Age						
< 12 months	166 (35.5)	504 (23.5)	1.8 (1.4–2.2)	< 0.001	1.4 (1.1–1.8)	0.009
≥ 12 months	301 (64.5)	1641 (76.5)	1			
Referred from another hospital						
Yes	282 (60.4)	1139 (53.1)	1.3 (1.1–1.7)	0.005	1.1 (0.9–1.4)	0.433
No	185 (39.6)	1006 (46.9)	1			
Surgery < 30 days on admission						
Yes	36 (7.7)	40 (1.9)	4.4 (2.8–7.0)	< 0.001	1.8 (1.0–3.3)	0.050
No	431 (92.3)	2105 (98.1)	1			
Severe sepsis or septic shock						
Yes	77 (16.5)	86 (4.0)	4.7 (3.4–6.6)	< 0.001	1.9 (1.3–2.9)	0.001
No	390 (83.5)	2059 (96.0)	1			
Use of urine catheter						
Yes	166 (35.6)	281 (13.1)	3.7 (2.9–4.6)	< 0.001	1.9 (1.3–2.6)	< 0.001
No	301 (64.4)	1864 (86.9)	1			
Use of central venous catheter						
Yes	64 (13.7)	49 (2.3)	6.8 (4.6–10.0)	< 0.001	1.8 (1.1–2.9)	0.024
No	403 (86.3)	2096 (97.7)	1			
Use of mechanical ventilation						
Yes	101 (21.6)	123 (5.7)	4.5 (3.4–6.0)	< 0.001	1.1 (0.7–1.7)	0.747
No	366 (78.4)	2022 (94.3)	1			

*HAI* healthcare-associated infections, *OR* odds ratio, *CI* confidence interval, *ICU* intensive care unit

Multivariable logistic regression analysis was done to determine independent risk factors of healthcare-associated infections

## Discussion

We explored the incidence and determinants of healthcare-associated infection to guide further preventative strategies. The cumulative incidence of healthcare-associated infection was higher compared to a previous study in the same setting after initiating a multifaceted intervention on infection control and antibiotic stewardship program [2]. The potential difficulties in sustaining the effect of multifaceted infection control interventions and antibiotic control programs may lead to increasing the rate of HAI in children in low- and middle-income countries. These include lack of performance feedback, staff turnover, news fatigue, distractions from other programs and initiatives, increased patient numbers, increased risk patient complexity, increased referred patients from outside hospitals, loosening of antibiotic prescribing standards over time, lack of consistency or intensity of input or reduction in frequency of training, lack of senior leadership input, never quite reaching the threshold of control of multi resistance organisms, and availability of resources such as hand hygiene and guidelines [11].

The important determinants of the high burden of healthcare-associated infection in children in this study were length of hospital stay more than 7 days, use of urinary catheter, use of central venous catheter, exposure to non-standardised antibiotics, aged less than 1 year, and presence of a severe sepsis or septic shock at the onset of diagnosis. These findings showed a general agreement with previous studies that have presented similar risk factors of healthcare-associated infection [1–10, 15].

Among extrinsic factors, length of hospital stay more than 7 days was found to be most highly associated with healthcare-associated infection in this study. It was consistent with previous published studies [1, 4, 9, 15, 16]. Longer hospital stay reflects the severity and complexity of the disease, requiring additional care, and additional time of exposure to various sources of infection [1, 5]. However, it is possible that length of hospital stay more than 7 days as a risk factor of healthcare-associated infection could be as a result of healthcare-associated infection itself. Lengths of hospital stay and healthcare-associated infections are interconnected, which make the cause-effect association difficult to determine.

Using central line catheter was considered as extrinsic risk factors of acquiring healthcare-associated infection [17]. When inserting the central catheter, hospital transient flora, which colonizes the patient’s skin, can enter the bloodstream, or bacteria on healthcare workers’ hands can be transmitted to the patient. Flora that colonises the patient’s skin, such as coagulase-negative staphylococci, can invade along the catheter from the insertion site, via a contaminated solution, or via the catheter connection. Minor skin abrasions can be an entry for pathogens. Not keeping central line entry points dry and clean, and taking too frequent blood samples may lead to increased risk for introduction of pathogens into these catheters [6]. When there are fewer adherences to aseptic technique during insertion and care of a central venous catheter, healthcare-associated infection potentially occurs [6].

Furthermore, the longer the duration of catheterization, the higher the risk of skin colonization by hospital flora is associated with increase the risk of catheter-related healthcare-associated infection [7]. In this study, central venous catheters were usually not changed until they were no longer indicated, or malfunctioned, or they became infected because inserting the catheter was technically difficult.

The use of central venous catheter could also relate to the severity of disease. It is assumed that patients using central venous catheter receive more medications and have more frequent access of their central venous catheters [10]. However, it was not feasible to gather that information in this study. To reduce the incidence of infection, it is therefore important to strictly apply maximal sterile and barrier precautions when inserting and maintaining central line catheter and performing surgery, including adherence to routine and proper hand hygiene practices, avoidance of contamination and adequate skin asepsis and dressings, reduce inappropriate use of invasive procedures, and remove catheter as soon as it is no longer needed [2].

The use of non-standardised antibiotics for community-acquired infections could be considered a risk factor of healthcare-associated infection, which is also a recognized risk factor for healthcare-associated infection related to antibiotic resistance bacteria [18–20]. A previous published study also revealed that the use of antibiotics more than 10 days in ICU could increase the risk for healthcare-associated infection by 5 times [9]. Therefore, antibiotic guidelines are needed and the compliance with those guidelines should be regularly assessed within everyday practices.

Children admitted to the hospital with severe sepsis or septic shock at the onset of diagnosis were defined as a critically ill condition. This kind of population frequently need a PICU admission as well as the use of invasive devices that lead to increasing the risk of developing hospital-acquired infections and death [21].

Preventive strategies that could be proposed to prevent healthcare-associated infections are sending patients home promptly when indicated, careful thought about whether central venous lines or urinary catheters are necessary, early removal of all invasive devices when no longer necessary, the rational use of antibiotics for suspected bacterial infections, isolating patient to prevent potentially healthcare-associated pathogen, and administering antibiotics directed for healthcare-associated infection when patients develop signs and symptoms of new infection during hospitalization.

Aged less than one year is an intrinsic factor that cannot be controlled in increasing the risk of developing healthcare-associated infection [16]. Even though the young age could not be changed, it can raise awareness if this is a risk factor and potentially mitigate harmful outcomes by altering the management of these infants.

Identified risk factors from this study could potentially be prevented to reduce the risk of acquiring healthcare-associated infection. Some are modifiable, and some are not based on the disease severity and treatment required. Knowing the exact indications for using invasive devices and daily assessing when to remove those devices would also reduce the necessity of prolonged use of invasive devices. Moreover, using rational antibiotics based on available guidelines would potentially reduce the risk of healthcare-associated infection.

The risk factors of healthcare-associated infection described in our study correspond to those in other published studies in low- and middle-income countries [1]. However, as far as we are aware, this was among the first studies on exploring risk factors of healthcare-associated infection in paediatric population. This study identifies several areas where infection control measures and antibiotic stewardship program need to be implemented.

The limitation of this study is only performed at a single teaching hospital. Therefore, findings from this study may not directly apply to other institutions with different patient populations and medical practices.

## Conclusions

In conclusion, the risk factors of healthcare-associated infection found in our study include length of hospital stay > 7 days, use of central venous and urinary catheter, use of non-standardized antibiotics, diagnosis of severe sepsis, and young age.

## Supplementary Information

**Additional file 1. ** The dataset of healthcare-associated infection.

## Data Availability

All data generated or analyzed during this study are included in this published article [and its Additional file]. The data used during the current study are also available from the corresponding author (IKM) on request.

## Abbreviations

CDC: Centers for disease control and prevention
CVL: Central venous line
HAI: Healthcare-associated infections
NHSN: National Healthcare Safety Network
NNIS: National Nosocomial Infections Surveillance
PICU: Paediatric intensive care unit
